# Does long-term object priming depend on the explicit detection of object identity at encoding?

**DOI:** 10.3389/fpsyg.2015.00270

**Published:** 2015-03-20

**Authors:** Carlos A. Gomes, Andrew Mayes

**Affiliations:** ^1^Human Memory Laboratory, School of Psychological Sciences, University of ManchesterManchester, UK; ^2^Department of Psychology, Saarland University, SaarbrückenGermany

**Keywords:** repetition priming, implicit memory, object identification, selective-attention, recognition memory, response learning

## Abstract

It is currently unclear whether objects have to be explicitly identified at encoding for reliable behavioral long-term object priming to occur. We conducted two experiments that investigated long-term object and non-object priming using a selective-attention encoding manipulation that reduces explicit object identification. In Experiment 1, participants either counted dots flashed within an object picture (shallow encoding) or engaged in an animacy task (deep encoding) at study, whereas, at test, they performed an object-decision task. Priming, as measured by reaction times (RTs), was observed for both types of encoding, and was of equivalent magnitude. In Experiment 2, non-object priming (faster RTs for studied relative to unstudied non-objects) was also obtained under the same selective-attention encoding manipulation as in Experiment 1, and the magnitude of the priming effect was equivalent between experiments. In contrast, we observed a linear decrement in recognition memory accuracy across conditions (deep encoding of Experiment 1 > shallow encoding Experiment 1 > shallow encoding of Experiment 2), suggesting that priming was not contaminated by explicit memory strategies. We argue that our results are more consistent with the identification/production framework than the perceptual/conceptual distinction, and we conclude that priming of pictures largely ignored at encoding can be subserved by the automatic retrieval of two types of instances: one at the motor level and another at an object-decision level.

## Introduction

It is a well-known phenomenon that a previous encounter with a stimulus can help the subsequent identification, production, and/or classification of the same or related stimulus. Such facilitation or bias in processing a repeated stimulus is commonly known as priming (Schacter, 1987; Tulving and Schacter, 1990), a type of implicit memory believed to occur even in the absence of any explicit knowledge of previous encounters with the studied items (e.g., Bowers and Schacter, 1990; Tulving et al., 1991).

A topic of intensive debate has been whether selective-attention and, relatedly, explicit stimulus identification during encoding are necessary for priming to occur. Evidence that conscious awareness is not a pre-requisite for successful priming performance comes from masked priming studies. In this paradigm, an item (e.g., the word “table”) is presented fully visible and preceded by a prime displayed for a sub-thresholded duration (e.g., 40 ms) and often sandwiched between a forward and backward pattern mask, effectively preventing its conscious identification. Critically, the prime can be identical to the target stimulus (e.g., “table”) or not (e.g., “window”). Performance, as measured, for example, by reaction times (RTs), is usually better when prime and target are of the same item rather than different items, suggesting that the prime is being unconsciously processed (e.g., Forster and Davis, 1984; Greenwald et al., 1996; Bodner and Masson, 1997; Masson and Isaak, 1999). However, masked priming effects are normally short-lived (the lag between prime and target must often not exceed a couple 100 ms, although see Duss et al., 2014 for a possible exception), suggesting that the cognitive mechanisms that govern this kind of priming are likely to be very distinct from those that support long-term priming (Henson, 2003), the latter of which is the focus of the current paper.

Apart from the masked priming paradigm, there are other ways to restrict stimulus processing by means of diverting attention away from the target item. Considerable effort has been expended in determining whether priming can occur independently of attention during study. One popular method is to instruct participants to process a given stimulus (e.g., reading target words) whilst performing a secondary, unrelated task on distractor stimuli (e.g., digit monitoring). Initial evidence using this approach provided mixed results, with some studies showing marked effects of divided-attention (e.g., Stone et al., 1998, 2000; Rajaram et al., 2001; Prull, 2013), while others showing little to no effect of dividing attention at study (e.g., Parkin and Russo, 1990; Szymanski and MacLeod, 1996; Light et al., 2000; Soldan et al., 2008). Mulligan and Hartman (1996) and Mulligan (1997) suggested that differences among studies could stem from the nature of the priming task administered during the test phase. A useful heuristic has been the division of tests into perceptual and conceptual (Blaxton, 1989; Roediger et al., 1992). Perceptual tests, such as lexical decision and word identification tasks, mainly require analysis of the physical features of the items, whereas conceptual tests, such as the category exemplar task, promote semantic analysis of stimuli. Some studies have reported divided-attention effects in conceptual but not perceptual tests (e.g., Parkin and Russo, 1990; Mulligan and Hartman, 1996; Szymanski and MacLeod, 1996; Mulligan, 1997, 1998; Soldan et al., 2008) leading to the idea that conceptual, but not perceptual, priming tests demand attentional resources at encoding.

More recently, however, several studies have also reported deleterious effects of dividing attention on perceptual word priming performance (e.g., Light and Prull, 1995; Stone et al., 1998; Mulligan and Hornstein, 2000; Mulligan, 2002; Berry et al., 2006). Some researchers have proposed that the identification/production framework (Gabrieli et al., 1999) better accounts for the effects of divided-attention. This framework distinguishes between production and identification priming, measured in tasks such as category exemplar verification and perceptual identification, respectively. In production tasks, the test cues trigger a response competition among several plausible solutions, and are believed to be impaired by manipulations of attention, whereas identification tasks are relatively unaffected. However, reports that production priming is unaffected by divided-attention (e.g., Light et al., 2000) and that identification priming is impaired (e.g., Light and Prull, 1995) have posed problems for this account as well.

Several other experimental variables have been proposed to be at the heart of the discrepancy among the aforementioned studies (see Spataro et al., 2011 for a review). For instance, it has been suggested that word priming may be affected by divided-attention to the extent that the distractor task interferes with word identification (Stone et al., 1998). This is most likely to occur in certain selective-attention paradigms, in which participants are instructed to attend to a distractor stimulus (or a feature of the target stimulus), whilst ignoring the target stimulus (or another feature of the same stimulus). Because the primary task does not require processing of the ignored item, stimulus identification is very unlikely to occur, or, if it does occur, it is greatly impoverished. Several studies have found that word priming was significantly reduced when participants were instructed to ignore the words but to attend to some other dimension of the words such as color (e.g., Stone et al., 1998, 2000; Rajaram et al., 2001) and abolished altogether if target words were ignored and participants had to focus on a distractor stimulus separated from the target words at encoding (e.g., Hawley and Johnston, 1991; MacDonald and MacLeod, 1998). Interestingly, in some of these studies (e.g., Mulligan and Hornstein, 2000; Stone et al., 2000; Rajaram et al., 2001), lexical identity of words was processed at study (as indicated, for example, by slower RTs for words than non-words during a color naming study task) but still led to a marked reduction in later perceptual priming. These results led to the idea that perceptual word priming may be dependent on several fundamental criteria over and above automatic lexical identity, such as conscious awareness of the identity of the study words during encoding (but see Mulligan and Hornstein, 2000; Rajaram et al., 2001 for an alternative explanation).

However, it remains uncertain how selective-attention, and, thus, the absence of explicit stimulus identification, at encoding affects perceptual non-word priming, since most selective-attention paradigms have mainly used word stimuli. As indicated above, processes related to conscious word identification may be critical for the emergence of subsequent perceptual word priming (but see Mulligan and Hornstein, 2000), but it may not be the case for non-word materials such as object pictures. In fact, given that pictorial stimuli are more novel, it is plausible that centrally presented pictures are sufficiently distinctive from one another to be uniquely encoded into a memory-supportive perceptual representation (Nelson, 1979), which could be independent of attentional manipulations. Although some studies did report selective-attention effects on later object picture priming, they either used a negative priming paradigm (e.g., Ballesteros et al., 2006) or presented target objects in ignored locations (e.g., Eger et al., 2004; Ballesteros and Mayas, 2014), so it is unclear whether object priming can occur after selective-attention encoding when attention is manipulated within dimensions of a centrally presented target object as opposed to across perceptual objects as in negative priming. Furthermore, it is plausible that the implicit perceptual system is selective, in the sense that it may only effectively represent task-relevant information (i.e., attended objects) but not ignored information (unattended objects).

Finally, it is also possible that some forms of response learning are independent of attention at encoding. Response learning, in the context of priming studies, refers to a special case of memory retrieval in which outcomes associated with the processing of a previously encountered stimulus are automatically retrieved upon stimulus repetition (e.g., Logan, 1990; Horner and Henson, 2008, 2009, 2011; Henson et al., 2014). Such stimulus–response (S–R) retrieval is believed to by-pass many of the laborious operations needed during the initial engagement with that stimulus, leading to faster RTs (and, thus, priming). These retrieved outcomes can be bound at multiple levels of representation such as at the motor level (stimulus-action bindings), at a decision level (e.g., Yes/No decision) or/and at a classification level (e.g., bigger/smaller judgment; e.g., Horner and Henson, 2009; Henson et al., 2014). For the present purposes, we questioned whether bindings at the stimulus-action level could partly contribute to the priming effects observed since participants used the same keys at both study and test phases.

We conducted two experiments that aimed to investigate the role of explicit object identification in a novel selective-attention encoding task using object picture stimuli. In Experiment 1, participants engaged in either a shallow or deep encoding task on object pictures whereas, at test, an object-decision task was administered. In Experiment 2, participants engaged in the same shallow encoding task as in Experiment 1 but only non-objects were presented.

To anticipate our key findings, we showed that even when explicit stimulus identification at encoding was unlikely, both object and non-object priming occurred during the object-decision task, and these priming effects had an equal magnitude. Priming appeared to have been largely driven by the retrieval of previous response mappings at the motor level, as it was greater when the same key had been pressed between study and test phases relative to when different keys were used. Nevertheless, residual priming was still observed for the different-key condition. In the General Discussion, we summarize the implication of these findings for current theories of priming.

## Experiment 1

The present experiment sought to determine whether priming for familiar objects could be obtained under selective-attention encoding conditions that do not promote explicit object identification. We designed a novel encoding task that diverted participants’ attention away from object meaning by having them perform an unrelated task (counting the number of red dots flashed within the object picture). It is important to note that during this task, attention is directed towards a change in the physical properties of the image (e.g., a change in pixel color) and not to a competing stimulus, like in negative priming studies. We also administered a task that involved making animacy judgments (deep condition) in order to be able to compare the magnitude of the priming effect between full-attention (animacy) and selective-attention (dot) encoding conditions. This comparison allowed us to determine whether fully expressed perceptual priming during the object-decision task can only be detected when objects are encoded under full-attention conditions. We predicted that positive perceptual priming should occur in this task regardless of encoding task, but we left open the question of whether the magnitude of priming for shallowly-encoded objects is reduced relative to priming for deeply-encoded objects. Finally, a recognition memory task was also employed on a separate group of participants, using the same study procedure as the participants assigned to the priming task.

### Methods

#### Participants

Forty-six undergraduate students enrolled in the Psychology course at the University of Manchester were recruited. All participants had normal or corrected-to-normal vision and gave informed written consent to take part in this study.

#### Materials

**FIGURE 1:**
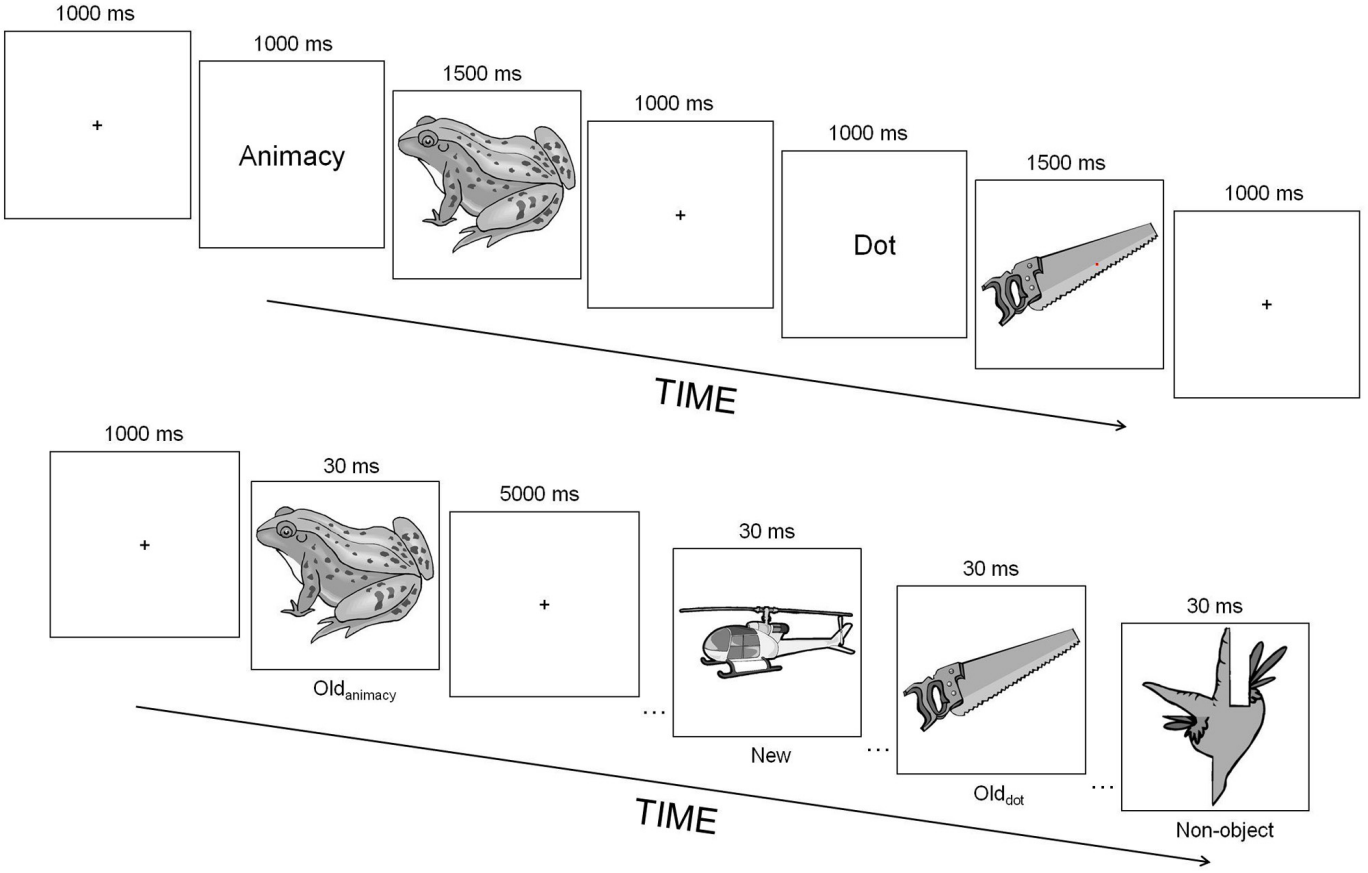
**Experimental paradigm of Experiment 1**. At study (top), participants either counted the number of red dots flashed within the object picture (dot task) or judged whether an object was either animate or inanimate (animacy task). At test (bottom), they decided whether a picture represented either an object or non-object (object-decision task). The numbers above each event correspond to the duration of those events in milliseconds. Old_animacy_, objects studied during the animacy task; Old_dot_, objects studied during the dot task; New, unstudied objects; Non-object, non-objects.

Sixty black-and-white pictures of common objects were selected from an online clipart database^1^. Shadows and other external features were removed from the pictures and all images were rescaled to fit in a box of 400 by 400 pixels. Half of the pictures comprised animate objects (e.g., animals) whereas the other half represented inanimate objects (e.g., vehicles). In addition, a different set of 30 pictures depicting new objects were altered to create the non-objects used in the test task (see **Figure 1** for an example, and Gomes and Mayes, 2014 for details about how these non-objects were created).

#### Procedure

**Figure 1** shows the experimental design used in this experiment. Participants engaged in two different tasks at study: in the “dot” task, they indicated whether they had seen either one or two red dots flashed within the picture whereas in the “animacy” task, they indicated whether a picture depicted an animate or inanimate object. The encoding task was randomly selected on each trial and a cue word was always shown before the presentation of a picture to inform participants which task they were about to perform. For each participant, 40 pictures out of the entire picture pool were randomly assigned to the animacy task (20) and to the dot (20) task, with the constraint that for each task, half of the pictures depicted animate objects whereas the other half inanimate objects. Each study trial began with a fixation cross shown for 1000 ms followed by the cue word, also presented for 1000 ms. A picture of an object was presented straight after the cue for 1500 ms. If the dot task was used, each dot was briefly flashed for 500 ms after picture onset and at a random location within the image. When two dots were presented, they were shown sequentially with an interval of ~200 ms between them, and the two dots always appeared at different random locations within the object picture. Accuracy was emphasized and participants were told to ignore the images, as attending to the images could interfere with task performance. They were also made aware that the dots could appear at any time during the image’s presentation, and were encouraged to scan the image thoroughly until it went off the screen. These instructions were used to ensure that participants were fully engaged in the dot task and to reduce the likelihood of explicit object processing and identification. Participants pressed one of the control keys on the keyboard for animate/one-dot decisions, and the other control key for inanimate/two-dots decisions. Immediately after the study phase, half of the participants were randomly selected to perform the priming task whereas the remaining half engaged in the recognition task. For the test phase of the priming task, 20 new pictures of objects as well as an additional 30 pictures of non-objects were shown along with the 40 studied objects (half of them had been presented during the dot task and the other half during the animacy task). Participants were instructed to decide as quickly and as accurately as possible whether a picture represented an object or a non-object (object-decision task). Each test trial was initiated by a fixation cross for 1000 ms followed by a brief presentation of either an object or a non-object for 30 ms. By using such short (but unmasked) presentation durations we encouraged participants to rely more on the perceptual properties of the images rather than their semantic meaning. Participants attempted to respond within 5000 ms. They pressed one of the control keys for object decisions, and the other control key for non-object decisions. For the recognition task, participants were instructed to decide as quickly and as accurately as possible whether an object had been presented at study. This and subsequent experiments were programmed and run using the Matlab^2^ toolbox Cogent^3^.

All experiments reported in this article were approved by the School of Psychological Sciences Research Ethics Committee of the University of Manchester.

#### Design and Analysis

The experimental design consisted of Condition (deep, shallow, new) as a within-subject factor and Test Task (priming, recognition) as a between-subject factor. The data were analyzed using (mixed) repeated measures analysis of variance (ANOVA) and *t*-tests on accuracy scores and RTs. A Huynh–Feldt correction was applied to the degrees of freedom of those tests for which the assumption of sphericity was violated. Proportional priming scores were also computed using the formula [(new-old)/new] for RTs in order to facilitate comparison between experiments by controlling for linear baseline differences between experiments (Schnyer et al., 2006); one-sample *t*-tests were used to compare these scores against zero. The alpha level was set, for all statistical tests, at 0.05.

### Results

RTs that were more than 2.5 SD above or below the mean value of each condition were considered outliers and removed from subsequent analyses. In addition, incorrect trials, defined as either an incorrect response or an absence of response during the object-decision task, were also removed from the analyses. These procedures resulted in the elimination of ~7% of trials; errors did not vary systematically across conditions.

#### Study Phase Results

The accuracy for both the animacy (0.88) and dot (0.69) tasks during the study phase of the priming experiment was significantly above chance [animacy: *t*(23) = 79.39, *p* < 0.001, *d* = 17.60; dot: *t*(23) = 22.31, *p* < 0.001, *d* = 4.60] suggesting that responding was more than mere guessing. Similar results were obtained for the study phase of the recognition memory task with the accuracy for both the animacy (0.89) and dot (0.68) tasks being above chance levels [animacy: *t*(21) = 67.26, *p* < 0.001, *d* = 14.08; dot: *t*(21) = 22.37, *p* < 0.001, *d* = 4.86].

#### Priming Results

**Table 1** shows the mean accuracy and RTs for each condition (deep, shallow, new and non-object) as well as the difference in accuracy levels between old and new objects, and proportional priming scores, calculated as the difference between old and new divided by new.

**Table 1:** Mean, difference in accuracy levels between old and new and proportional reaction time (RTs; [new-old]/new) data during the object-decision task for Experiments 1 and 2.

	Mean		Difference	Proportion
	Acc	RTs	Acc	RTs
**Experiment 1**
Old_animacy_	0.96 (0.02)	569 (14)	0.04 (0.01)	0.04 (0.01)
Old_dot_	0.95 (0.02)	570 (15)	0.02 (0.01)	0.04 (0.01)
New	0.92 (0.02)	594 (17)		
Non-objects	0.92 (0.01)	711 (32)		
**Experiment 2**
Old_dot_	0.94 (0.01)	702 (30)	-0.002 (0.02)	0.03 (0.01)
New	0.94 (0.02)	723 (34)		
Objects	0.95 (0.01)	638 (18)		

*Standard error of the mean is given within parenthesis. Experiment 1: Old_animacy_, objects studied during the animacy task; Old_dot_, objects studied during the dot task; New, unstudied objects; Non-objects, non-objects. Experiment 2: Old_dot_, non-objects studied during the dot task; New, unstudied non-objects; Objects, objects.*

*T*-tests on the difference in accuracy levels between old and new objects revealed a significant effect for both deep, *t*(23) = 3.48, *p* = 0.001, *d* = 0.67, and shallow, *t*(23) = 2.41, *p* < 0.05, *d* = 0.50, conditions, which did not differ between them, *t*(23) = 0.95, *p* > 0.10, *d* = 0.14. The same pattern of results was obtained for proportional RT data, with both deep and shallow objects being judged significantly faster than new objects [deep vs. new: *t*(23) = 2.55, *p* < 0.01, *d* = 0.54, shallow vs. new: *t*(23) = 2.88, *p* < 0.01, *d* = 0.62], but not differing between them, *t*(23) = –0.03, *p* > 0.10, *d* = 0.01. Thus, priming was obtained for both deep and shallow conditions when either accuracy or RTs were used as a behavioral measure, and the magnitude of these effects did not differ between the study conditions.

We next enquired whether these priming effects were driven by more elaborate analysis of the stimulus (i.e., conceptual priming) or, alternatively, by more perceptual processing (i.e., perceptual priming). For that purpose, we divided the trials within each condition (deep, shallow, and new) into two separate groups (fast and slow) based on a RT median split. This analysis is predicated on the idea that, behaviorally, RTs during conceptual priming would take longer than RTs during perceptual priming, as the result of participants retrieving additional information regarding some conceptual properties of the objects. Thus, if the priming effects reported above were solely due to the influence of slower trials (which, presumably, contain more conceptual processing), then priming should be obtained for the slower group, whereas the mean RT difference between fast old and fast new trials should be identical, leading to a significant Group × Condition interaction. A 2 Group (Fast, Slow) × 3 Condition (Deep, Shallow, New) repeated measures ANOVA revealed an unsurprising significant main effect of group, *F*(1,23) = 255.12, *p* < 0.001, $\eta_{p}^{2}$ = 0.92, as well as a more interesting significant main effect of condition, *F*(1,23) = 6.53, *p* < 0.01, $\eta_{p}^{2}$ = 0.22, with faster responses for both deeply- and shallowly- encoded objects relative to new objects. Importantly, the interaction was not significant, *F*(1,23) = 1.47, *p* > 0.10, $\eta_{p}^{2}$ = 0.06, indicating that both the faster and slower studied objects had shorter RTs than the faster and slower new objects, respectively (see **Table 2**).

**Table 2:** Mean RTs for fast and slow trials (based on a median-split) for Experiments 1 and 2.

	Fast	Slow
**Experiment 1**
Old_animacy_	497 (11)	636 (17)
Old_dot_	501 (13)	635 (18)
New	518 (13)	665 (21)
**Experiment 2**
Old_dot_	599 (24)	799 (36)
New	611 (26)	830 (45)

*Standard error of the mean is given within parenthesis. Experiment 1: Old_animacy_, objects studied during the animacy task; Old_dot_, objects studied during the dot task; New, unstudied objects. Experiment 2: Old_dot_, non-objects studied during the dot task; New, unstudied non-objects.*

Next we investigated the impact of response learning on the priming effects obtained above. As we explained in the Introduction, it is possible that S–R bindings at the stimulus-action level could have contributed to the priming effect when the same keys were used in both study and test phases. In order to test this possibility we divided each study condition into two groups. One group contained trials in which the same key was pressed during both study and test phases (same-key group), whereas the other group consisted of trials in which the different keys were pressed between experimental phases (different-key group). **Table 3** shows the data for this analysis. Proportional priming scores were calculated for each study condition and submitted to a 2 Response (Same-key, Different-key) × 2 Condition (Deep, Shallow) repeated measures ANOVA. This analysis only revealed a main effect of response, *F*(1,22) = 11.82, *p* < 0.01, $\eta_{p}^{2}$ = 0.35, with greater priming for same-key (0.05) than different-key (0.02) trials. Collapsed across conditions, same-key priming was significant, *t*(22) = 3.15, *p* < 0.01, *d* = 0.71, whereas different-key priming approached significance, *t*(21) = 1.56, *p* = 0.065, *d* = 0.38.

**Table 3:** Mean RTs and proportional priming ([new-old]/new) for same-key (Same) and different-key (Different) trials for Experiments 1 and 2.

	Same		Different	
	RTs	Proportion	RTs	Proportion
**Experiment 1**
Old_animacy_	561 (14)	0.05 (0.02)	575 (15)	0.03 (0.02)
Old_dot_	564 (16)	0.04 (0.01)	580 (15)	0.02 (0.01)
**Experiment 2**
Old_dot_	697 (29)	0.03 (0.02)	709 (32)	0.02 (0.01)

*Standard error of the mean is given within parenthesis. Experiment 1: Old_animacy_, objects studied during the animacy task; Old_dot_, objects studied during the dot task; New, unstudied objects. Experiment 2: Old_dot_, non-objects studied during the dot task; New, unstudied non-objects.*

Although our results suggest that explicit object identification is not critical for priming to occur, it could be argued that priming was obtained for shallowly-encoded objects because of the influence of those trials in which object identification was more likely to occur during study. If that was the case, then it is reasonable to assume that priming would be greater in participants who disengaged from the primary dot task in favor of object processing (i.e., participants with poor study-task accuracy) than in participants who were fully engaged (i.e., participants with high study-task accuracy). We performed correlational analyses between average study accuracy and priming performance separately for deeply- and shallowly- encoded objects. If participants with lowest accuracy at study (possibly due to task disengagement) showed greater priming than those with higher study accuracy, it would suggest that object identification at study probably aided later priming. However, neither correlation coefficient was significant [deep: *r*(24) = 0.11, *p* > 0.10; shallow: *r*(24) = 0.26, *p* > 0.10], suggesting that greater effort/engagement during the study phase was not a likely contributor for the emergence of the priming effects above.

Because the above result is based on a null result, we decided to test the effects of study engagement on priming performance on an item-by-item basis. For each study condition, we divided items into two groups based on whether participants had made a correct or an incorrect judgment at study. Because there were not sufficient incorrect trials for an accurate analysis for the deep encoding condition, only the shallow encoding condition was included in this analysis. Priming was significant regardless of whether items had been correctly judged at study (0.03), *t*(22) = 2.55, *p* < 0.01, *d* = 0.57, or not (0.04), *t*(22) = 2.16, *p* < 0.05, *d* = 0.44, and the difference between study-correct and study-incorrect priming did not reach significance, *t*(22) = –0.33, *p* > 0.10, *d* = 0.07, indicating that study-task engagement was not a determinant factor in the emergence of priming.

Finally, some research suggests that animate objects are identified more accurately and faster than inanimate objects (e.g., Laws and Neve, 1999; Laws, 2000). We reasoned that if participants were indeed trying to identify the objects during the dot task, then we should observe faster RTs for animate relative to inanimate objects, as the result of participants covertly detecting the animacy category to which each object belonged. We computed the difference in RTs between animate and inanimate objects presented during each encoding condition, excluding outlying scores that were below or above 2.5 SD. Predictably, the RT difference between animate and inanimate objects for the animacy encoding condition (29 ms) was significant, *t*(21) = 2.20, *p* < 0.05, *d* = 0.48, whereas the RT difference between animate and inanimate objects for the dot encoding condition (–2 ms) was not significant, *t*(21) = –0.22, *p* > 0.10, *d* = 0.04. The difference between the two scores also reached significance, *t*(20) = 1.92, *p* < 0.05, *d* = 0.42.

Based on these results, we, therefore, conclude that priming in the shallow condition was not dependent on the likelihood of object identity processing at encoding.

#### Recognition Results

One participant responded incorrectly to all the new trials but one and, for that reason, was excluded from the following analyses.

**Table 4** shows the proportion of responses and mean RTs for each response category (hits, misses, correct rejections, and false alarms).

**Table 4:** Proportion of responses (Pr; range within parenthesis) and RTs (standard error of the mean within parenthesis) for each response category for Experiments 1 and 2.

	Experiment 1		Experiment 2	
Category	Pr	RTs	Pr	RTs
H_animacy_	0.76 (0.03)	1022 (42)	–	–
M_animacy_	0.24 (0.03)	1141 (98)	–	–
H_dot_	0.40 (0.03)	1184 (100)	0.37 (0.03)	1262 (55)
M_dot_	0.60 (0.03)	1020 (59)	0.63 (0.03)	1275 (69)
CR	0.89 (0.02)	962 (48)	0.76 (0.03)	1284 (72)
FA	0.11 (0.02)	1340 (137)	0.24 (0.03)	1442 (97)

*H_animacy_, hit rate for objects encoded during the animacy task; M_animacy_, misses for objects encoded during the animacy task; H_dot_, hit rate for objects encoded during the dot task; M_dot_, misses for objects encoded during the dot task; CR, correct rejections; FA, false alarm rate.*

We first computed the corrected hit rate (hit rate minus false alarm rate) for each condition separately (deep and shallow). A paired *t*-test revealed a significant effect, *t*(21) = 11.00, *p* < 0.001, *d* = 2.40, indicating higher recognition accuracy for deeply-encoded (0.64) than shallowly-encoded (0.29) objects. However, both conditions were significantly above chance levels [deep: *t*(20) = 18.47, *p* < 0.001, *d* = 4.06; shallow: *t*(20) = 10.29, *p* < 0.001, *d* = 2.23].

Given that participants’ memory scores were above chance in both study conditions, the RT data from the object-decision task were contrasted with the RT data from the recognition task in order to ascertain whether recognition could have driven the priming effects obtained above. If RTs during the recognition task were at least as fast as those during the priming task, then the possibility that participants used explicit memory strategies to aid priming performance would be conceivable. However, if priming RTs were substantially faster than those during recognition memory, the contamination account would be unlikely. A 2 Task (Priming, Recognition) × 2 Condition (Deep, Shallow) mixed repeated measures ANOVA only revealed a significant main effect of condition, *F*(1,43) = 4.85, *p* < 0.05, $\eta_{p}^{2}$ = 0.10, with faster RTs for deeply- relative to shallowly-encoded objects, as well as a main effect of task, *F*(1,43) = 71.34, *p* < 0.001, $\eta_{p}^{2}$ = 0.62, suggesting much slower responses during the recognition task than during the priming task. More importantly, the interaction factor was also significant, *F*(1,43) = 4.73, *p* < 0.05, $\eta_{p}^{2}$ = 0.10, reflecting the fact that whereas shallowly- and deeply- encoded objects were equally fast in the priming task, shallowly-encoded objects had slower RTs than deeply-encoded objects in the recognition memory task. Independent *t*-tests revealed that recognition judgments in both study conditions were significantly slower than judgments during the priming task (both *p* < 0.001), even when the slowest priming responses were contrasted with the fastest recognition memory responses.

### Discussion

The main purpose of the present experiment was to determine whether long-term object priming could be obtained under selective-encoding conditions that discourage explicit object identification. Our results were clear in this respect. Priming, as measured by RTs during an object-decision task, was obtained when participants had either made animacy judgments (deep encoding task) or counted the number of red dots briefly flashed within an image (shallow encoding task), suggesting that explicit identification was not the determinant factor in order to observe long-term object priming. Critically, the magnitude of the priming effect seemed to be both qualitatively and quantitatively equivalent between the two encoding conditions, suggesting a seemingly common underlying source. Recognition memory was also measured in a separate group of participants using the exact same encoding procedure, but altering test instructions. Although recognition memory was above chance levels for both deeply- and shallowly- encoded objects, shallowly-encoded objects were more poorly remembered than deeply-encoded ones. Furthermore, RTs during the recognition memory task were substantially slower than those during the priming task, even when the fastest recognition memory responses were compared with the slowest priming responses. This latter finding suggests that our priming effects were unlikely to have been contaminated by explicit memory processes.

The critical question is, however, what generated the priming effects observed in the present experiment. It is clear that a substantial portion of the priming effect of both deeply- and shallowly- encoded objects was due to response retrieval at the stimulus-action level, given that studied objects that required the same key press at both study and test phases produced greater priming than objects requiring different key presses. The fact that shallowly-encoded objects were affected by this response retrieval mechanism is remarkable considering that the responses generated during the dot task were based on an external perceptual attribute (i.e., dots) rather than on object processing *per se*. In typical long-term object priming studies examining response learning, the studied objects are generally encoded under full-attention conditions and participants are required to make a decision regarding certain semantic properties of the objects (like in our deep encoding task). The fact that stimulus-action links can form even when the response does not involve explicit object processing indicates the power of response retrieval during binary-response tasks (e.g., Horner and Henson, 2009, 2011; Dennis and Perfect, 2013; Henson et al., 2014).

Even so, response retrieval alone cannot explain the modest different-key priming obtained. One possibility is that priming was influenced by perceptual factors that were kept constant throughout the experiment; that is, the same picture was presented at both study and test phases, so priming could have resulted from the recapitulation of perceptual processes. This would be consistent with the transfer-appropriate processing (TAP) framework, which proposes that memory performance is determined by the overlap of processes at test that were initially engaged at encoding (Morris et al., 1977).

Even though appealing, explaining shallowly-encoded object priming in terms of a processing account appears problematic. In order to perform the object-decision task correctly, object identification is obviously required. Undoubtedly, during the deep encoding task, explicit object identification was not only probable but necessary in order to perform the task correctly. However, during the dot task, participants need not consciously detect what the picture represented, as their sole task was counting dots. We carefully instructed participants that the objects should be ignored since attending to them could affect their performance in the dot task. Furthermore, we also made participants aware that the dots could appear at any time during the picture’s presentation, and, therefore, they should look for the dots until the image disappeared from the screen. This means that, even if object identification did occur, it was likely to have been incidental to the task aims, uncommon and much less effective than for the objects studied under deep encoding conditions. Thus, according to a purely processing account, perceptual priming should have been at least greatly reduced in relation to priming for deeply-encoded objects. We did not find this. We also do not believe that the lack of a statistical difference between the two encoding conditions was due to a Type II error, because we have recently obtained very similar results (i.e., equivalent priming for shallowly- and deeply- encoded objects) using a similar procedure in a different participant sample (Gomes et al., 2015).

Nevertheless, to confirm that explicit object identification is unnecessary for object priming to take place (at least, using the study/test procedure employed in this study), one must be able to show that priming can occur for stimuli that cannot be identified under the conditions imposed by our experimental paradigm. This was the motivation for conducting Experiment 2.

## Experiment 2

Experiment 1 showed that behavioral priming can be obtained under impoverished encoding conditions that do not promote explicit object identification. However, it is still possible that objects were identified; especially considering that recognition memory under the same shallow encoding conditions was still above chance. Nevertheless, identification during the recognition memory test may have been only partial (e.g., the general shape of the object, or a particular salient feature), which could have been sufficient to support above-chance recognition memory levels. In the present experiment, we tested whether priming can be obtained even when object identification is extremely unlikely to have occurred. We decided to use the non-objects from the test phase of Experiment 1 (as opposed to, for example, abstract shapes) in the study phase of the present experiment since these non-objects lack meaning but are perceptually similar to the objects utilized in Experiment 1 (e.g., similar line complexity, spatial frequencies), making the two experiments more comparable. At test, participants performed either the object-decision task or a recognition memory test.

### Methods

#### Participants

Forty-four undergraduate students enrolled in the Psychology course at the University of Manchester were recruited. All participants had normal or corrected-to-normal vision and gave informed written consent to take part in this study. Four participants were excluded; one showed extreme difficulties in performing the tasks and three due to a technical error.

#### Materials, Procedure, and Design

The materials, procedure, and design used in this experiment were identical to those described for Experiment 1 with the following exceptions. First, there was a total of 60 images of non-objects and 30 pictures of objects. Second, 30 non-objects were presented at study whereas at test, these 30 studied non-objects were shown along with 30 new non-objects as well as 30 pictures of objects. Third, we decided to administer only the dot task at encoding, as we were concerned that if participants were required to identify the non-objects during the animacy task, they would try to identify them also during the dot task. Finally, in order to increase non-object accuracy to a level similar to that observed for objects in Experiment 1, non-objects were presented for 200 ms during the object-decision task.

### Results

RTs that were more than 2.5 SD above or below the mean value of each condition were considered outliers and removed from subsequent analyses. In addition, incorrect trials, defined as either an incorrect response or an absence of response during the object-decision task, were also removed from the analyses. This resulted in the elimination of ~9.5% of trials; errors did not vary systematically across conditions.

#### Non-Object Identification

Since each non-object created for Experiment 1 consisted of parts of a real object which were rearranged into a new image configuration, it is possible that the identity of these non-objects was perceived either during the dot or the object-decision task (e.g., participants could have internally reassembled the different parts of objects into their original configuration). In order to determine the degree of object identification during either the study or test phase we conducted two preliminary control experiments that measured identification accuracy. Eight participants that did not take part in any of the experiments reported in this paper performed two tests. In the first of these tests, we instructed participants to perform the dot task, and, if possible, to write the name of the object after the dot decision. It was stressed that the dot task was the primary task and that they should not neglect it in favor of object identification (this was done to ensure that accuracy levels were not markedly different from those during the study phase of the real experiment). It should be pointed out, however, that these instructions will likely lead to an overestimation of identification accuracy since participants are asked to overtly identify the non-object while engaging in the dot task (i.e., a divided-attention manipulation). In contrast, participants in the shallow encoding condition of the present experiment (and of Experiment 1) are instructed to perform the dot task whilst ignoring the object picture (i.e., a selective-attention manipulation). Despite the possibility of overestimation, participants’ non-object identification accuracy was indeed very poor (*M* = 0.38, SD = 0.32). In the second test, we presented the non-objects very quickly on the screen (at the same duration as in the real experiment) and asked participants to indicate whether they could identify and write the name of the object. Again, non-object identification was extremely poor (*M* = 0.32, SD = 0.33).

#### Study Phase Results

Accuracy was above chance levels during the study phase of both the priming [*M* = 0.64, *t*(20) = 22.81, *p* < 0.001, *d* = 4.92] and recognition memory [*M* = 0.64, *t*(23) = 26.12, *p* < 0.001, *d* = 5.33] tasks. Furthermore, accuracy for the present study data did not differ reliably from the accuracy for the study data of Experiment 1, *t*(43) = 1.07, *p* > 0.10, *d* = 0.33, suggesting that no more effort was applied in the identification of non-objects relative to the objects in Experiment 1.

#### Priming Results

The difference in accuracy levels between old and new objects and proportional RT priming were calculated for each participant (see **Table 1**). Although there was no effect of accuracy, *t*(19) = 0.10, *p* > 0.10, *d* = 0.03, proportional RT priming reached significance, *t*(19) = 2.29, *p* < 0.05, *d* = 0.60. This priming effect did not differ from the one in Experiment 1 when either the dot task, *t*(42) = –0.70, *p* > 0.10, *d* = 0.22, or the animacy task, *t*(42) = 0.52, *p* > 0.10, *d* = 0.16, was used at encoding. Thus, priming was obtained for shallowly-encoded non-objects and this priming effect did not differ from the one obtained when pictures of objects were used. Because the critical argument is that object priming can be obtained despite the absence of explicit object identification, we computed proportional priming for only those non-objects which were unidentified by all participants in the control task described above (mean: 9 trials; range: 6–13 trials). Even when the identity of non-objects was extremely unlikely, proportional non-object priming remained highly significant (0.04), *t*(19) = 2.96, *p* < 0.01, *d* = 0.80.

Like with Experiment 1, we split the shallow and new conditions into a fast and slow group based on the median RT of each specific condition to determine whether priming was specific to slower trials, which could suggest the influence of slower (e.g., semantic) processes (see **Table 2**). A 2 Group (Fast, Slow) × 2 Condition (Shallow, New) repeated measures ANOVA revealed a main effect of group, *F*(1,19) = 100.66, *p* < 0.001, $\eta_{p}^{2}$ = 0.84, as well as a main effect of condition, *F*(1,19) = 4.86, *p* < 0.05, $\eta_{p}^{2}$ = 0.20. Planned comparisons revealed that shallow objects had significantly shorter RTs than new objects in the slow group, *t*(19) = –1.91, *p* < 0.05, *d* = 0.42, and a trend for significance for the faster responses, *t*(19) = –1.49, *p* = 0.077, *d* = 0.32. The interaction did not approach significance, *F*(1,19) = 1.28, *p* > 0.10, $\eta_{p}^{2}$ = 0.06.

Next, we examined whether response retrieval could have partly been responsible for the speed-up observed for the shallowly-encoded non-objects, since there was evidence in Experiment 1 that a substantial portion of the priming effect for shallowly-encoded objects was due to the retrieval of bindings coding motor responses (see **Table 3**). Even though same-key priming (0.03) was numerically larger than different-key (0.02) priming, the difference between them did not reach significance, *t*(19) = 0.70, *p* > 0.10, *d* = 0.13. Different-key priming was significant, *t*(19) = 1.75, *p* < 0.05, *d* = 0.50, and same-key priming approached significance, *t*(19) = 1.44, *p* = 0.08, *d* = 0.33^4^. A 2 Experiment (Experiment 1, Experiment 2) × 2 Response (Same-key, Different-key) mixed repeated measures ANOVA only revealed a trend for a main effect of response, *F*(1,42) = 3.23, *p* = 0.079, $\eta_{p}^{2}$ = 0.07, with greater priming for same-key (0.04) than different-key (0.02) trials. Nevertheless, when collapsed across experiments, priming for both types of response were significant [same-key: *t*(43) = 3.24, *p* = 0.001, *d* = 0.50; different-key: *t*(43) = 1.93, *p* < 0.05, *d* = 0.33]. The interaction did not approach significance, *F*(1,42) = 0.48, *p* > 0.10, $\eta_{p}^{2}$ = 0.01.

Finally, the correlation between study accuracy and priming performance did not approach significance, *r* = 0.37, *p* > 0.10, and there was no difference between study-correct and study-incorrect priming, *t*(19) = 0.96, *p* > 0.10, *d* = 0.20.

#### Recognition Results

The recognition data for this experiment are shown on **Table 4**.

A one-sample *t*-test on the corrected hit rate revealed a significant effect, *t*(23) = 5.91, *p* < 0.001, *d* = 1.27, indicating above-chance recognition memory (0.14). However, recognition memory was more accurate for shallowly-encoded objects in Experiment 1 than for shallowly-encoded non-objects in Experiment 2, *t*(43) = –4.22, *p* < 0.001, *d* = 3.75.

Also, RTs of studied non-objects during the recognition memory test were substantially slower than RTs of studied non-objects during the priming test, *t*(34.78) = 8.43, *p* < 0.001, *d* = 2.86, suggesting that priming effects in the present experiment were unlikely to have been contaminated by the use of explicit memory strategies.

### Discussion

The main result of the present experiment was the observation of a priming effect even when the stimuli used comprised largely unidentifiable non-objects. Proportional priming scores did not differ reliably between Experiment 1 and the present experiment, and there was no evidence that slower, higher-level processes were involved in this kind of priming, as suggested by similar priming for faster and slower trials.

However, because each non-object used in the present experiment was created by rearranging the parts of a real object into a new configuration, it could be argued that participants attempted to identify the non-object (during either the dot task or the object-decision task) by mentally rearranging the different parts into the original configuration. In two control experiments, we showed that participants’ non-object identification accuracy was very low for either the dot task or the object-decision task, even though identification was likely to have been overestimated during these control experiments. Thus, priming could not have arisen solely on the basis of non-object identification. Even if a few non-objects were spontaneously identified during the encoding phase, we find it extremely unlikely that they could have produced a net priming effect. Although reliable, priming was still small in magnitude, so we would not expect that a few identified non-objects would be capable of overpowering the negative influence of the majority of studied non-objects that, presumably, failed to show any priming-related speed-up. One could still make the point that we did not obtain stronger priming effects because the studied, but unidentified, non-objects introduced noise into our priming measure and, consequently, reduced the priming effect. However, if non-object priming had really resulted only from those non-objects that had been identified at study, then we should have seen a substantial difference in the magnitude of the priming effect for deeply-encoded objects in Experiment 1 relative to the shallowly-encoded non-objects in the present experiment, since all deeply-encoded objects had been surely identified. This was clearly not the case, as both types of priming were equivalent in magnitude. Note also that RT differences for new items cannot account for the pattern of results between experiments because we used proportional priming scores, which account for linear baseline effects (Horner and Henson, 2009). Finally, non-object priming was reliable even when we analyzed only non-objects that were not identifiable during the control task.

The fact that non-object priming in the present experiment was obtained under the same study and test conditions as object priming in Experiment 1, and that the same experimental manipulations had similar effects on both kinds of priming (e.g., equal for fast and slow trials, greater for same- vs. different-key trials), suggests that explicit object identification did not underlie priming in either experiment, at least when measured during the object-decision task. These results strongly indicate that both non-object and object priming are likely to share a common source.

## General Discussion

Two experiments were conducted to determine whether explicit object identification is necessary to produce reliable priming effects. In Experiment 1, participants performed both a deep encoding task under full-attention conditions (animacy task) and a shallow encoding task under selective-attention conditions (dot counting) on different object pictures. At test, a group of participants engaged in an object-decision task whereas another group performed a recognition memory test. Priming, as measured by RTs, was obtained for both deeply- and shallowly- encoded objects and the magnitude of the effect did not differ between these conditions. We also measured recognition memory on a separate group of participants using the same encoding procedure but altering test instructions. Although recognition memory was above chance levels for both conditions, it was greatly reduced for shallowly-encoded objects, and recognition memory RTs were substantially slower than RTs during the priming task. In Experiment 2, we presented non-objects at encoding while participants performed the same dot task as in Experiment 1. Priming (faster RTs for studied relative to unstudied non-objects) during the object-decision task was equivalent to that of Experiment 1. For both experiments, a substantial portion of the priming effect could be explained by the repetition of the motor action (i.e., same key press), although residual priming was still observed when different keys were pressed between study and test phases. Finally, contrary to the equivalent priming between experiments, recognition memory was substantially reduced in Experiment 2 relative to Experiment 1 and RTs were much faster during priming than recognition.

The generally greater priming that was found when the same rather than different keys were used at both study and test phases highlights the importance of considering S–R associations in priming experiments. Note that the dot task in particular did not require any processing of stimuli where the dots appeared, which is consistent with some evidence from the negative priming literature suggesting that S–R bindings are encoded even under conditions of inattention (e.g., Rothermund et al., 2005; Frings et al., 2007).

Reports of priming driven by the repetition of motor responses (also called stimulus-action bindings) have been published (e.g., Dobbins et al., 2004; Horner and Henson, 2009; Dennis and Perfect, 2013) but they are not undisputed (e.g., Logan, 1990; Schnyer et al., 2007; Dennis et al., 2010). One possibility as to why stimulus-action bindings played such a prominent role in our experiments is that no other explicit response was associated with the stimulus itself (e.g., a decision about some semantic property of the object). It is possible that the effects of stimulus-action bindings on priming performance are more likely to be observed when other mnemonic sources are ambiguous or absent. Indeed, recent research suggests that multiple S–R bindings are coded in distinct, but possibly inter-connected, brain structures (e.g., Race et al., 2009; Horner and Henson, 2012; Henson et al., 2014), converging in response-production brain regions, and, ultimately, generating a motor response. If the different S–R signals do interact (Horner and Henson, 2009, 2011; Dennis et al., 2010; Dennis and Perfect, 2013), then stronger S–R bindings, such as stimulus-classification or stimulus-decision bindings, may overpower the weak influence of stimulus-action associations. On the other hand, if the only kind of S–R binding available are stimulus-action bindings (as seems to be the case at least for the dot task) their effects on behavior will be accentuated. Future research will be needed to verify this idea.

Another interesting finding was that both object and non-object priming seemed sensitive to S–R associations in a similar way. However, the finding of reliable non-object priming driven by learned S–R associations contrasts with recent evidence from a study that failed to find reliable long-term S–R learning for novel objects. In this study, Saggar et al. (2010) asked participants to perform a classification task on novel objects consisting of 2-D abstract line-based shapes. At study, they performed a classification task (deciding whether an object was fat) on shapes presented either once, twice or three times. At test, studied shapes presented three times were shown along with non-studied shapes and participants performed either the same task as at study or the reverse task (i.e., deciding whether an object was slim). For the present purposes, the authors observed that objects that had been studied three times were not judged faster than unstudied objects during the test phase, and they did not find decision-switch costs when the test cue was reversed (a typical signature of stimulus-decision learning). In a subsequent experiment, however, the authors showed that real objects did show the expected behavioral decrement in long-term priming performance when the decision cue was changed between study and test phases.

It is not clear why Saggar et al. (2010) did not find long-term non-object priming whereas we did. We should note, however, that there are a number of discrepancies between the studies that may limit comparability. First, for the experiment with novel shapes, Saggar et al. (2010) used a relatively difficult task that relied on subjective classifications (e.g., something classified as slim will likely vary across participants) whereas, for the experiment with real objects, they used a more typical size-judgment task (deciding whether an object is bigger/smaller than a shoebox). If the novel-shape priming measure was noisier, it would have masked effects that were probably already small in magnitude. In contrast, our paradigm has the advantage that the same study and test task (and associated instructions) can be used, and only the type of stimulus is altered, thus, making the two experiments more comparable. Second, the authors used relatively basic shapes of objects, whereas our non-objects had the same perceptual complexity as that of real objects. As the authors concluded based on their computational modeling data, absence of reliable long-term priming for novel objects may relate to interference caused from weak representations of perceptually similar shapes.

Despite playing a central role in the current priming effects, S–R learning did not seem to be the only factor at play. We still observed a form of priming that was independent of key-press congruency, as suggested by modest different-key priming in both Experiments 1 and 2. One possibility, which would be consistent with processing accounts (Blaxton, 1989; Roediger et al., 1992), is that this residual priming for deeply- and shallowly- encoded objects resulted from the recapitulation of encoding-related processes at the time of testing. Because the object-decision task involves processes related to object identification, one would expect that study-related identification processes would be reactivated. Although this may well be the case for deeply-encoded objects (i.e., participants need to identify an object in order to make animacy judgments), explicit object identification was unlikely to have occurred during the dot study task. Even if it did occur, such processing was probably much reduced relative to the processing of deeply-encoded objects, which should have resulted in a poorer match between study and test phase processes, and, consequently, a reduction in priming for shallowly-encoded objects.

We believe that our results are more consistent with the identification/production view. There is some evidence that the negative effects of manipulating attention at encoding are most pronounced during production than identification tasks (Gabrieli et al., 1999), although this remains somewhat controversial (e.g., Light et al., 2000; Spataro et al., 2010; Prull, 2013). The general idea is that the test cues in production tasks (e.g., word-stem completion) are more likely to initiate response competition amongst multiple plausible alternatives (e.g., CARROT and CARPET are valid completions for the word stem CAR___). In contrast, the test cues in identification tasks guide the retrieval of unique and unambiguous responses, so attentional manipulations at encoding do not affect identification priming. In the present study, there was only one appropriate, unambiguous response during the object-decision task (a picture could represent either an object or a non-object) so there was no involvement of response competition, and, thus, no more attention was required at encoding. This may explain why we found equivalent priming between deeply- and shallowly- encoded objects.

We should point out that we are not arguing that perceptual object priming is never sensitive to attentional manipulations and/or object identification at encoding. In fact, it will be important for future research to determine whether certain manipulations will differentially affect priming for shallowly- and deeply- encoded objects and, thus, lead to attentional effects. For instance, some research has identified certain forms of long-term priming that can last several weeks or even years (e.g., Mitchell and Brown, 1988; Mitchell, 2006). It is, thus, possible that priming for deeply-encoded objects subsists even when longer delays separate study and test phases, whereas priming for shallowly-encoded objects may be reduced or even eliminated by similar time lags.

It remains unanswered, however, what kind of information was used during test that facilitated responding in the object-decision task for different-key trials. We propose that an object-status instance may have been implicitly encoded during the study task and associated with each object presented at study. For example, because only objects were shown during the study phase of Experiment 1 (and participants were aware of this fact) an “object label” could have been incidentally conceived for each and every individual study object. At test, repetition of an object triggered the automatic retrieval of this encoded label, making information regarding object status readily accessible and, thus, shortening RTs. The same reasoning applies to the non-objects in Experiment 2: a “non-object label” could have been formed at encoding, since only non-objects were shown, and retrieval of this label facilitated responding for studied non-objects during the object-decision task. The implication of this view is that priming should be reduced when the signals derived from the retrieval of these labels become ambiguous, and undermine their utility in aiding priming performance during the object-decision task. Although not easily testable using the present paradigm, an alternative could be to ask participants to rate the valence of words shown on top of the ignored object picture, such that each object would be associated with a negative word in one trial and a positive word in another. Later, an affective priming test could be administered for the picture stimuli. Since the retrieved valence labels associated with each object would be ambiguous, we would expect reduced priming under these conditions relative to when either only positive or only negative words were shown at study.

If our view is correct, it could be regarded as an extension to the identification/production account because it would suggest that response competition generated unconsciously (i.e., the automatic retrieval of ambiguous encoded labels) can also affect priming, whereas when such competition is absent (like in the present study) priming remains relatively unaffected by attention manipulations.

Although our proposal remains speculative, we have put forth a similar idea in a recent study, in which participants rated the meaningfulness of sentences associating two unrelated objects (Gomes and Mayes, 2014). Associative priming (faster RTs for intact relative to recombined pairs) was obtained during a size-judgment task only when intact pairs had been encoded with meaningful sentences. Importantly, the sentences never explicitly referred to size information, which means that the information that supported associative priming must have stemmed from an inferential process at the time of encoding. That is, rating a meaningful sentence such as “The elephant was transported by the train” is diagnostic of item-level relative size (e.g., since the train is bigger than the elephant, it can transport the elephant); such inference may have been automatically encoded and probably retrieved upon repetition of an intact pair. It should be noted, however, that the participants in the Gomes and Mayes’s experiments were explicitly required to attend to the objects and engaged in a relatively high-level elaborative encoding task. The current data extends our previous findings by suggesting that encoded decisions can also be automatically established without explicit object processing at encoding.

It should be pointed out that the interpretation above still relies on motor- and unconscious decision-bindings being associated with some form of perceptual representation. Because reliable priming was obtained for non-identifiable non-objects, we believe that this perceptual representation may encode “partial” identification (e.g., representation of salient parts of an object). This representation may allow the binding of motor and decision associations, but may not be sufficient to support object identification. It could be argued then that reactivation of visual information encoded in this perceptual representation could potentially also be responsible for the non-motor priming effect, which would be consistent with a processing account. Note, however, that simply extracting perceptual information from partial representations that do not mark object identity would be of little use during the object-decision task, because such representations would not contain information regarding object possibility.

## Conclusion

In the present report, both object and non-object priming were obtained when explicit object identification at study was disrupted by means of a selective-attention manipulation. Our results highlighted the importance of response learning at the motor level and indicate that object priming for ignored stimuli may also be dependent on the automatic establishment and retrieval of unconsciously-encoded decision instances. An interesting question for future research is whether these instances are stored in the typical brain regions that subserve other forms of long-term priming. It will also be important to validate our argument that explicit object identification is not a requirement for object priming to occur. This could be done by incorporating complementary techniques such as eyetracking, which could be used to check, for example, the amount of fixations to diagnostic parts of objects and/or to measure the extent to which participants actively scan each individual picture.

## Conflict of Interest Statement

The authors declare that the research was conducted in the absence of any commercial or financial relationships that could be construed as a potential conflict of interest.

## Footnotes

1 http://www.clipart.com/

2 http://www.mathworks.com

3 http://www.vislab.ucl.ac.uk/cogent.php

4 Although same-key priming did not quite reach significance in the present experiment, this was due to an outlier that was over 2.5 SD below mean proportional priming. Without this outlier the mean proportional priming rose from 0.029 to 0.041 and was significantly above chance, *t*(18) = 1.44, *p* = 0.01, *d* = 0.59, and modestly larger than different-key priming, *t*(18) = 1.38, *p* = 0.09, *d* = 0.32. However, this participant was not an outlier in other contrasts of interest so removing it from the current analysis would be circular.

## References

[B1] Ballesteros S. Mayas J. (2014). Selective attention affects conceptual object priming and recognition: a study with young and older adults. *Front. Psychol.* 5:1567 10.3389/fpsyg.2014.01567 PMC4290485 25628588

[B2] Ballesteros S. Reales J. M. García E. Carrasco M. (2006). Selective attention affects implicit and explicit memory for familiar pictures at different delay conditions. *Psicothema* 18 88–99. 17296015

[B3] Berry C. J. Henson R. N. A. Shanks D. R. (2006). On the relationship between repetition priming and recognition memory: insights from a computational model. *J. Mem. Lang.* 55 515–533 10.1016/j.jml.2006.08.008

[B4] Blaxton T. A. (1989). Investigating dissociations among memory measures - support for a transfer-appropriate processing framework. *J. Exp. Psychol. Learn. Mem. Cogn.* 15 657–668 10.1037/0278-7393.15.4.657

[B5] Bodner G. E. Masson M. E. J. (1997). Masked repetition priming of words and nonwords: evidence for a nonlexical basis for priming. *J. Mem. Lang.* 37 268–293 10.1006/jmla.1996.2507

[B6] Bowers J. S. Schacter D. L. (1990). Implicit memory and test awareness. *J. Exp. Psychol. Learn. Mem. Cogn.* 16 404–416 10.1037/0278-7393.16.3.404 2140400

[B7] Dennis I. Carder H. Perfect T. J. (2010). Sizing up the associative account of repetition priming. *Psychol. Res.* 74 35–49 10.1007/s00426-008-0224-9 19142658

[B8] Dennis I. Perfect T. J. (2013). Do stimulus–action associations contribute to repetition priming? *J. Exp. Psychol. Learn. Mem. Cogn.* 39 85–95 10.1037/a0028479 22582963

[B9] Dobbins I. G. Schnyer D. M. Verfaellie M. Schacter D. L. (2004). Cortical activity reductions during repetition priming can result from rapid response learning. *Nature* 428 316–319 10.1038/nature02400 14990968

[B10] Duss S. B. Reber T. P. Hänggi J. Schwab S. Wiest R. Müri R. M. (2014). Unconscious relational encoding depends on hippocampus. *Brain* 137 3355–3370 10.1093/brain/awu270 25273998 PMC4240286

[B11] Eger E. Henson R. N. A. Driver J. Dolan R. J. (2004). BOLD repetition decreases in object-responsive ventral visual areas depend on spatial attention. *J. Neurophysiol.* 92 1241–1247 10.1152/jn.00206.2004 15056686

[B12] Forster K. L. Davis C. (1984). Repetition priming and frequency attenuation in lexical access. *J. Exp. Psychol. Learn. Mem. Cogn.* 10 680–698 10.1037/0278-7393.10.4.680

[B13] Frings C. Rothermund K. Wentura D. (2007). Distractor repetitions retrieve previous responses to targets. *Q. J. Exp. Psychol.* 60 1367–1377 10.1080/17470210600955645 17853245

[B14] Gabrieli J. D. E. Vaidya C. J. Stone M. Francis W. S. Thomson-Schill S. L. Fleischman D. A. (1999). Convergent behavioral and neuropsychological evidence for a distinction between identification and production forms of repetition priming. *J. Exp. Psychol. Gen.* 128 479–498 10.1037/0096-3445.128.4.479 10650584

[B15] Gomes C. A. Mayes A. (2014). The kinds of information that support novel associative object priming and how these differ from those that support item priming. *Memory* 10.1080/09658211.2014.937722 [Epub ahead of print]. 25051200

[B16] Gomes C. A. Montaldi D. Mayes A. (2015). The pupil as an indicator of unconscious memory: introducing the pupil priming effect. *Psychophysiology* 10.1111/psyp.12412 [Epub ahead of print]. 25656874

[B17] Greenwald A. G. Draine S. C. Abrams R. L. (1996). Three cognitive markers of unconscious semantic activation. *Science* 273 1699–1702 10.1126/science.273.5282.1699 8781230

[B18] Hawley K. J. Johnston W. A. (1991). Long-term perceptual memory for briefly exposed words as a function of awareness and attention. *J. Exp. Psychol. Hum. Percept. Perform.* 17 807–815 10.1037/0096-1523.17.3.807 1834792

[B19] Henson R. N. A. (2003). Neuroimaging studies of priming. *Prog. Neurobiol.* 70 53–81 10.1016/S0301-0082(03)00086-8 12927334

[B20] Henson R. N. Eckstein D. Waszak F. Frings C. Horner A. J. (2014). Stimulus-response bindings in priming. *Trends Cogn. Sci.* 18 376–383 10.1016/j.tics.2014.03.004 24768034 PMC4074350

[B21] Horner A. J. Henson R. N. (2008). Priming, response learning and repetition suppression. *Neuropsychologia* 46 1979–1991 10.1016/j.neuropsychologia.2008.01.018 18328508 PMC2430995

[B22] Horner A. J. Henson R. N. (2009). Bindings between stimuli and multiple response codes dominate long-lag repetition priming in speeded classification tasks. *J. Exp. Psychol. Learn. Mem. Cogn.* 35 757–779 10.1037/a0015262 19379048

[B23] Horner A. J. Henson R. N. (2011). Stimulus-response bindings code both abstract and specific representations of stimuli: evidence from a classification priming design that reverses multiple levels of response representation. *Mem. Cogn.* 39 1457–1471 10.3758/s13421-011-0118-8 PMC3205272 21671105

[B24] Horner A. J. Henson R. N. (2012). Incongruent abstract stimulus-response bindings result in response interference: FMRI and EEG evidence from visual object classification priming. *J. Cogn. Neurosci.* 24 760–773 10.1162/jocn-a-00163 22066586 PMC3601413

[B25] Laws K. R. (2000). Category-specific naming errors in normal subjects: the influence of evolution and experience. *Brain Lang.* 75 123–133 10.1006/brln.2000.2348 11023642

[B26] Laws K. R. Neve C. (1999). A “normal” category-specific advantage for naming living things. *Neuropsychologia* 37 1263–1269 10.1016/S0028-3932(99)00018-4 10530726

[B27] Light L. L. Prull M. W. (1995). Aging, divided attention, and repetition priming. *Swiss J. Psychol.* 54 87–101.

[B28] Light L. L. Prull M. W. Kennison R. F. (2000). Divided attention, aging, and priming in exemplar generation and category verification. *Mem. Cogn.* 28 856–872 10.3758/BF03198421 10983460

[B29] Logan G. D. (1990). Repetition priming and automaticity: common underlying mechanisms? *Cogn. Psychol.* 22 1–35 10.1016/0010-0285(90)90002-L

[B30] MacDonald P. A. MacLeod C. M. (1998). The influence of attention at encoding on direct and indirect remembering. *Acta Psychol.* 98 291–310 10.1016/S0001-6918(97)00047-4 9621835

[B31] Masson M. E. Isaak M. I. (1999). Masked priming of words and nonwords in a naming task: further evidence for a nonlexical basis for priming. *Mem. Cogn.* 27 399–412 10.3758/BF03211536 10355231

[B32] Mitchell D. B. (2006). Nonconscious priming after 17 Years: invulnerable implicit memory? *Psychol. Sci.* 17 925–929 10.1111/j.1467-9280.2006.01805.x 17176420

[B33] Mitchell D. B. Brown A. S. (1988). Persistent repetition priming in picture naming and its dissociation from recognition memory. *J. Exp. Psychol. Learn. Mem. Cogn.* 14 213–222 10.1037/0278-7393.14.2.213 2967344

[B34] Morris C. D. Bransford J. D. Franks J. J. (1977). Levels of processing versus transfer appropriate processing. *J. Verbal Learn. Verbal Behav.* 16 519–533 10.1016/S0022-5371(77)80016-9

[B35] Mulligan N. W. (1997). Attention and implicit memory tests: the effects of varying attentional load on conceptual priming. *Mem. Cogn.* 25 11–17 10.3758/BF03197281 9046866

[B36] Mulligan N. W. (1998). The role of attention during encoding in implicit and explicit memory. *J. Exp. Psychol. Learn. Mem. Cogn.* 24 27–47 10.1037/0278-7393.24.1.27 9438952

[B37] Mulligan N. W. (2002). Attention and perceptual implicit memory: effects of selective versus divided attention and number of visual objects. *Psychol. Res.* 66 157–165 10.1007/s00426-002-0089-2 12192444

[B38] Mulligan N. W. Hartman M. (1996). Divided attention and indirect memory tests. *Mem. Cogn.* 24 453–465 10.3758/BF03200934 8757494

[B39] Mulligan N. W. Hornstein S. L. (2000). Attention and perceptual priming in the perceptual identification task. *J. Exp. Psychol. Learn. Mem. Cogn.* 26 626–637 10.1037/0278-7393.26.3.626 10855421

[B40] Nelson D. L. (1979). “Remembering pictures and words: appearance, significance, and name,” in *Levels of Processing in Human Memory,* eds Cermak L. S. Craik F. I. M. (Hillsdale, NJ: Lawrence Erlbaum), 45–76.

[B41] Parkin A. J. Russo R. (1990). Implicit and explicit memory and the automatic/effortful distinction. *Eur. J. Cogn. Psychol.* 2 71–80 10.1080/09541449008406198

[B42] Prull M. W. (2013). Attention and repetition priming in the verb generation task. *Acta Psychol.* 143 218–226 10.1016/j.actpsy.2013.03.010 23624574

[B43] Race E. A. Shanker S. Wagner A. D. (2009). Neural priming in human frontal cortex: multiple forms of learning reduce demands on the prefrontal executive system. *J. Cogn. Neurosci.* 21 1766–1781 10.1162/jocn.2009.21132 18823245 PMC2788302

[B44] Rajaram S. Srinivas K. Travers S. (2001). The effects of attention on perceptual implicit memory. *Mem. Cogn.* 29 920–930 10.3758/BF03195754 11820751

[B45] Roediger H. L. Weldon M. S. Stadler M. L. Riegler G. L. (1992). Direct comparison of two implicit memory tests: word fragment and word stem completion. *J. Exp. Psychol. Learn. Mem. Cogn.* 18 1251–1269 10.1037/0278-7393.18.6.1251 1447550

[B46] Rothermund K. Wentura D. De. Houwer J. (2005). Retrieval of incidental stimulus-response associations as a source of negative priming. *J. Exp. Psychol. Learn. Mem. Cogn.* 31 482–495 10.1037/0278-7393.31.3.482 15910132

[B47] Saggar M. Miikkulainen R. Schnyer D. M. (2010). Behavioral, neuroimaging, and computational evidence for perceptual caching in repetition priming. *Brain Res.* 1315 75–91 10.1016/j.brainres.2009.11.074 20005215

[B48] Schacter D. L. (1987). Implicit memory: history and current status. *J. Exp. Psychol. Learn. Mem. Cogn.* 13 501–518 10.1037/0278-7393.13.3.501 3160813

[B49] Schnyer D. M. Dobbins I. G. Nicholls L. Davis S. Verfaellie M. Schacter D. L. (2007). Item to decision mapping in rapid response learning. *Mem. Cogn.* 35 1472–1482 10.3758/BF03193617 PMC2034352 17948070

[B50] Schnyer D. M. Dobbins I. G. Nicholls L. Schacter D. L. Verfaellie M. (2006). Rapid response learning in amnesia: delineating associative learning components in repetition priming. *Neuropsychologia* 44 140–149 10.1016/j.neuropsychologia.2005.03.027 15893343

[B51] Soldan A. Mangels J. A. Cooper L. A. (2008). Effects of dividing attention during encoding on perceptual priming of unfamiliar visual objects. *Memory* 16 873–895 10.1080/09658210802360595 18821167 PMC2574786

[B52] Spataro P. Cestari V. Rossi-Arnaud C. (2011). The relationship between divided attention and implicit memory: a meta-analysis. *Acta Psychol.* 136 329–339 10.1016/j.actpsy.2010.12.007 21257140

[B53] Spataro P. Mulligan N. Rossi-Arnaud C. (2010). Effects of divided attention in the word-fragment completion task with unique and multiple solutions. *Eur. J. Cogn. Psychol.* 22 18–45 10.1080/09541440802685979

[B54] Stone M. Ladd S. L. Gabrieli J. D. E. (2000). The role of selective attention in perceptual and affective priming. *Am. J. Psychol.* 113 341–358 10.2307/1423363 10997232

[B55] Stone M. Ladd S. L. Vaidya C. J. Gabrieli J. D. (1998). Word-identification priming for ignored and attended words. *Conscious. Cogn.* 7 238–258 10.1006/ccog.1998.0326 9690028

[B56] Szymanski K. F. MacLeod C. M. (1996). Manipulation of attention at study affects an explicit but not an implicit test of memory. *Conscious. Cogn.* 5 165–175 10.1006/ccog.1996.0010 8978529

[B57] Tulving E. Hayman C. A. Macdonald C. A. (1991). Long-lasting perceptual priming and semantic learning in amnesia: a case experiment. *J. Exp. Psychol. Learn. Mem. Cogn.* 17 595–617 10.1037/0278-7393.17.4.595 1832430

[B58] Tulving E. Schacter D. L. (1990). Priming and human memory systems. *Science*, 247 301–306 10.1126/science.2296719 2296719

